# Ultrathin Ti_3_C_2_T_*x*_ (MXene) Nanosheet-Wrapped NiSe_2_ Octahedral Crystal for Enhanced Supercapacitor Performance and Synergetic Electrocatalytic Water Splitting

**DOI:** 10.1007/s40820-019-0261-5

**Published:** 2019-04-04

**Authors:** Hanmei Jiang, Zegao Wang, Qian Yang, Luxi Tan, Lichun Dong, Mingdong Dong

**Affiliations:** 1 Interdisciplinary Nanoscience Center (iNANO), Aarhus University, 8000 Aarhus-C, Denmark; 2 School of Chemistry and Chemical Engineering, Key Laboratory of Low-grade Energy Utilization Technologies and Systems of the Ministry of Education, Chongqing University, Chongqing, 400044 People’s Republic of China; 3 College of Materials Science and Engineering, Sichuan University, Chengdu, 610065 People’s Republic of China; 4 College of Chemistry and Molecular Engineering, Peking University, Beijing, 100871 People’s Republic of China

**Keywords:** MXene, NiSe_2_, Supercapacitor, Water splitting

## Abstract

Metal selenides, such as NiSe_2_, have exhibited great potentials as multifunctional materials for energy storage and conversation. However, the utilization of pure NiSe_2_ as electrode materials is limited by its poor cycling stability, low electrical conductivity, and insufficient electrochemically active sites. To remedy these defects, herein, a novel NiSe_2_/Ti_3_C_2_T_*x*_ hybrid with strong interfacial interaction and electrical properties is fabricated, by wrapping NiSe_2_ octahedral crystal with ultrathin Ti_3_C_2_T_*x*_ MXene nanosheet. The NiSe_2_/Ti_3_C_2_T_*x*_ hybrid exhibits excellent electrochemical performance, with a high specific capacitance of 531.2 F g^−1^ at 1 A g^−1^ for supercapacitor, low overpotential of 200 mV at 10 mA g^−1^, and small Tafel slope of 37.7 mV dec^−1^ for hydrogen evolution reaction (HER). Furthermore, greater cycling stabilities for NiSe_2_/Ti_3_C_2_T_*x*_ hybrid in both supercapacitor and HER have also been achieved. These significant improvements compared with unmodified NiSe_2_ should be owing to the strong interfacial interaction between NiSe_2_ octahedral crystal and Ti_3_C_2_T_*x*_ MXene, which provides enhanced conductivity, fast charge transfer as well as abundant active sites, and highlight the promising potentials in combinations of MXene with metal selenides for multifunctional applications such as energy storage and conversion.

**Electronic supplementary material:**

The online version of this article (10.1007/s40820-019-0261-5) contains supplementary material, which is available to authorized users.

## Highlights

- A strong interfacial chemical interaction between the NiSe_2_ nanocrystal and Ti_3_C_2_T_*x*_ MXene nanosheet (NiSe_2_/Ti_3_C_2_T_*x*_) was established by wrapping NiSe_2_ octahedral crystal with ultrathin Ti_3_C_2_T_*x*_ MXene nanosheet.
- NiSe_2_/Ti_3_C_2_T_*x*_ hybrid exhibits excellent performance and cycling stability both in supercapacitor and hydrogen evolution reaction.
- NiSe_2_ nanocrystals are stabilized by the covering of Ti_3_C_2_T_*x*_ MXene nanosheet which served as a protective layer from its oxidation.

**Electronic supplementary material:**

The online version of this article (10.1007/s40820-019-0261-5) contains supplementary material, which is available to authorized users.

## Graphical abstract

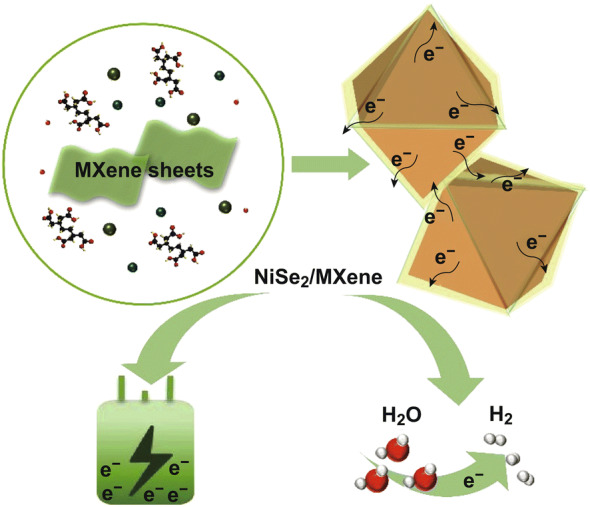

## Introduction

With the development of society, the energy crisis has become more and more prominent; thus, developing strategies toward high efficient energy storage and energy conversion has caught much attention [1, 2]. Owing to its high power density, long cycling life, and rapid charge–discharge rates, supercapacitors (SCs) have stand out as one of the most promising candidates for energy storage [3]. Hydrogen, as a clean and renewable energy, is considered as a promising candidate to overcome the environmental issues. Recently, the hydrogen generation through hydrogen evolution reaction (HER) is considered as one most cost-optimal energy conversion technique [4]. Nevertheless, to date, the state-of-the-art materials for electrochemical processes in energy storage or conversion, such as the SCs or HER, are still mostly based on noble metal-based materials [5, 6]. The rare storage and high cost of these materials severely restrict their practical applications. Therefore, non-noble metal-based materials with high capacitance and low activation energy for energy storage and renewable energy are profoundly needed to replace noble metal materials [7–9]. Moreover, developing a material with both energy storage and conversion could not only save the cost but also facilitate its integration. Recently, it has been reported that transitional metal oxides [10], hydroxides [11] and their hybrids [12, 13] have been widely investigated demonstrating their potential application in both energy storage and conversion. However, the low electronic conductivity and poor cycling stability still limit their application [14].

By contrast, transition metal selenides, with their high electrochemical activity as well as excellent thermal stability, might be a possible substitute for noble metal materials [15–18]. In particular, nickel selenides (NiSe_2_) not only have exhibited considerable potential in lithium-ion batteries [19–21], SCs [22–25], electrocatalyst [26–28] and photovoltaic [29], but also provided efficient HER function as the electrode materials [30]. Previous research suggested that the good electrochemical activity of nickel selenides should be owing to the unique electronic structure and multiple oxidation states. However, pure NiSe_2_ exhibits unsatisfying cycling stability, low electrical conductivity, and insufficient electrochemically active sites [31]. Therefore, hybriding with new materials is considered as the promising method to overcome the drawback.

Carbonaceous materials such as carbon nanowires [32] and reduced graphene oxide (rGO) [33, 34] are considered favorable hybridizers for enhancing the conductivity, yet their intrinsic nature usually limits the capacities for energy storage [35, 36]. Therefore, developing new conductivity materials beyond carbonaceous materials for future energy storage and producing renewable energy poses a major challenge. As recently reported, MXene, a new family of two-dimensional (2D) transitions, metal carbides, carbonitrides with the general formula of M_*n*+1_X_*n*_T_*x*_ (M is an early transition metal, X is C/N, and T_*x*_ is surface terminal groups such as hydroxyl (–OH) and fluorine (–F), etc.) have been introduced [37, 38]. In possession of the metallic conductivity with hydrophilic nature, which is seldom realized by many other 2D materials such as layered metal sulfides and graphene [39–41], MXenes have exhibit promising potential in lithium batteries and supercapacitors when hybridized as supporting materials [42–45]. For producing renewable energy, MXene with C_3_N_4_, Co-BDC MOF or MoS_2_ also displays superb electrocatalytic activity [46–48]. Nevertheless, MXene hybrid systems are rarely investigated. To our best of knowledge, their combinations with metal selenides have not yet been reported. In this work, a novel electrode material based on NiSe_2_ octahedral crystal wrapped with ultrathin Ti_3_C_2_T_*x*_ MXene nanosheet was prepared via a simple one-pot hydrothermal route, where the hybridized NiSe_2_/Ti_3_C_2_T_*x*_ exhibits unique multifunction property in energy storage and conversion. The results show that the hybridized NiSe_2_/Ti_3_C_2_T_*x*_ displays excellent performances with capacity of 531.2 F g^−1^ at 1 A g^−1^ for supercapacitors, which is among the highest performances of the modified metal selenides [49, 50], and it also exhibits relatively smaller Tafel slope of 37.7 mV dec^−1^ for HER, which is close to the value of Pt. Further investigations have reviewed that these high performances of NiSe_2_/Ti_3_C_2_T_*x*_ are owing to the unique structure of the hybrid in which the Ti_3_C_2_T_*x*_ MXene is evenly wrapped on the surface of the NiSe_2_ octahedral crystal via an interfacial interaction. Compared with pure NiSe_2_, such connection between the two compositions not only provides much faster charge transfer, but also increases durability as well.

## Experimental Section

### Reagent and Materials

Ti_3_AlC_2_ (98%) was purchased from the Forsman Scientific Co., Ltd. (Beijing, China). Polyvinylidene fluoride (≥ 99.5%) was purchased from Micxy Chemical Co., Ltd. (Chengdu, China). Nickel form (110 mesh per inch) was purchased from Chuan Dong Chemical Co., Ltd. (Chongqing, China). Carbon black was purchased from Cabot Corporation (Boston, USA). Hydrochloric acid (HCl, ACS grade, 36-38%), lithium fluoride (LiF, ACS grade, ≥ 99%), *n*-methyl-2-pyrrolidone (ACS grade, ≥ 99.8%), NiCl_2_ (ACS grade, ≥ 99%), Se powder (ACS grade, ≥ 99%), KOH (ACS grade, ≥ 99%), and EDTA-Na_2_ (ACS grade, ≥ 99%) were purchased from Sigma-Aldrich. All chemical materials were used as received without further purification.

### Synthesis of Ti_3_C_2_T_*x*_ Nanosheets

Ti_3_C_2_T_*x*_ nanosheets were prepared according to previous literature, by the selective etching of the Al layer of Ti_3_AlC_2_ using a mixture of concentrated HCl and LiF [41]. Briefly, 2 g of LiF was slowly added and dissolved into 20 mL of 9 mol L^−1^ HCl under stirring to prepare the etching solution. Then, 2 g of Ti_3_AlC_2_ powders was carefully added to the solution over the course of 10 min to avoid overheating. The reaction mixture was heated to 40 °C for 48 h, and then the resulting solid was thoroughly washed using deionized (DI) water for more than three times until the pH value of the supernatant liquid reached 6–7 after the centrifugation. The final Ti_3_C_2_T_*x*_ powder was obtained after drying the raw materials at 60 °C under vacuum for 12 h. And then, 100 mg of Ti_3_C_2_T_*x*_ powder was added to 10 mL of water. After sonication 1 h under Ar bubbling, the Ti_3_C_2_T_*x*_ sheet solution (10 mg mL^−1^) was formed and stored in fridge for further using.

### Synthesis NiSe_2_/Ti_3_C_2_T_*x*_ Hybrid

The NiSe_2_/Ti_3_C_2_T_*x*_ hybrid was synthesized by a one-pot hydrothermal method [27, 51]. Briefly, 2 mmol Se powder was firstly dissolved by 5 mL KOH solution (20 mol L^−1^) to form a brownish red solution. Subsequently, 1 mmol NiCl_2_ was dissolved in 19 mL DI water and mixed with 1.6 mmol EDTA-2Na (as chelating agent) to form a blue solution. Then 1 mL Ti_3_C_2_T_*x*_ sheets solution (10 mg mL^−1^) was added into the blue solution and ultrasonically dispersed for 30 min and then dropwisely added into the above brownish red solution. The as-prepared solution was transferred into a 50-mL Teflon-lined autoclave and heated at 180 °C for 24 h. After the reaction finished, the precipitates were collected by centrifugation and washed with DI water for several times until the pH value of the supernatant liquid reaches 7. Finally, the NiSe_2_/Ti_3_C_2_T_*x*_ hybrid was obtained by vacuum drying at 60 °C for 12 h. For comparison, unmodified NiSe_2_ was synthesized by following the same method but without adding Ti_3_C_2_T_*x*_ nanosheets.

### Characterization

X-ray diffraction (XRD) analyses were performed on a Rigaku D/MAX-r diffractometer with Cu Kα radiation (*λ* = 0.1541 nm). Raman spectra were measured using a Renishaw via Raman microscope with the excitation laser line at 514 nm. X-ray photoelectron spectra (XPS) were recorded on a Kratos Axis Ultra DLD spectrometer equipped with a monochromatic Al Kα X-ray source (75–150 W), and C 1*s* (284.8 eV) was used to calibrate all the XPS peaks before comparison. Scanning electron microscopy (SEM, NOVA-667) and transmission electron microscopy (TEM, Talos FEI) were used to study the morphology of the samples. High-angle annular dark-field scanning transmission electron microscopy (HAADF-STEM, Talos) was used to identify the elemental composition of the samples.

### Electrochemical Measurements

Both supercapacitor and HER performance of the as-synthesized NiSe_2_/Ti_3_C_2_T_*x*_ hybrid were evaluated at room temperature in the three-electrode system on a CHI660E workstation. For supercapacitor measurement, to prepare the working electrode, NiSe_2_/Ti_3_C_2_T_*x*_ or NiSe_2_ powders (80 wt%), carbon black (10 wt%), and polyvinylidene fluoride (10 wt%) were dispersed in *n*-methyl-2-pyrrolidone, and then the resulted slurry was coated onto a piece of nickel form (1.0 × 1.0 cm^2^), which was dried at 120 °C under vacuum for 12 h and pressed under 10 MP for 1 min to obtain the working electrode. The mass loading of NiSe_2_/Ti_3_C_2_T_*x*_ on the nickel foam was about 8 mg. The Ag/AgCl electrode and platinum wire were used as the reference and counter electrodes, respectively. The electrochemical measurements were performed in 2 M KOH aqueous solution. The cyclic voltammetry (CV) was carried out in the potential range of − 0.2 to 0.55 V versus Ag/AgCl electrode at a scan rate from 10 to 100 mV s^−1^. The galvanostatic charge–discharge (GCD) was also performed in the potential range of − 0.2 to 0.55 V versus Ag/AgCl electrode. The electrochemical impedance spectroscopy (EIS) was performed in the range of 10 mHz to 100 kHz with potential amplitude of 10 mV. The specific capacitance in the three-electrode system was calculated from the GCD according to Eq. 1 [52].

1 $$C_{\text{s}} = \frac{I\Delta t}{m\Delta V }$$

where *C*_s_ (F g^−1^) is the specific capacitance, *I* (A) is the discharge current, $\Delta t$ (s) is the discharge time, *m* (g) is the total active material mass on the electrode, and $\Delta V$ (V) is potential window during the discharge process.

For electrocatalytic reaction, 6 mg of active material (NiSe_2_/Ti_3_C_2_T_*x*_ hybrid, NiSe_2_, Ti_3_C_2_T_*x*_) and 15 μL of Nafion solution were added into 160 μL mixed solvent of deionized water and isopropanol with a volume ratio of 5:3. The dispersive process lasted for 1 h by sonication to form a homogeneous ink. Then, 1.5 μL of the ink was loaded onto a 3-mm-diameter glassy carbon electrode. The whole electrochemical measurement process was in 0.5 M H_2_SO_4_ electrolyte, using a graphite as counter electrode, an Ag/AgCl electrode as reference electrode, and the above glassy carbon containing catalyst film as the working electrode. All measured potentials versus Ag/AgCl were transferred to reversible hydrogen electrode (RHE) based on the Nernst equation: *E*(RHE) = *E*(Ag/AgCl) + 0.0592 pH + 0.197. The linear scan voltammogram (LSV) curves were obtained by sweeping potential from 0 to − 0.7 V versus Ag/AgCl with a scan rate of 5 mV s^−1^. EIS was carried out under the similar parameter settings as supercapacitor. The electrical double-layer capacitance (C_dl_) of as-prepared electrodes was obtained using cyclic voltammograms (CVs) in a non-faradaic potential range (0.4 to 0.5 V vs. RHE). The stability test was measured by the continuous CV with scan rate of 100 mV s^−1^ for 2000 times. After cycling, the polarization curve was measured again. The chronoamperometry current density–time curve was measured in a constant potential of − 0.25 V versus RHE.

## Result and Discussion

### Synthesis and Characterizations of NiSe_2_ and Ti_3_C_2_T_*x*_/NiSe_2_

**Scheme 1:**
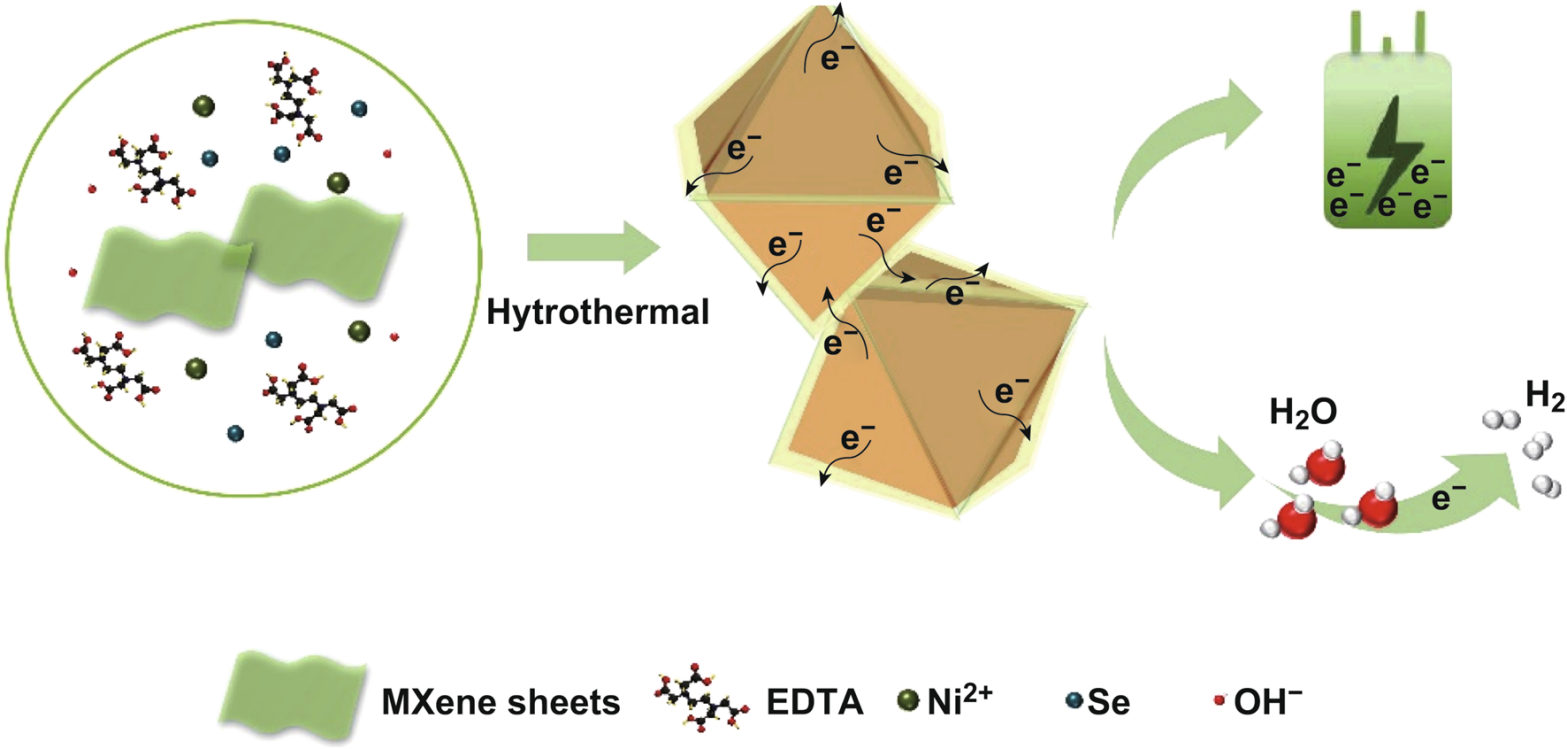
Schematic illustration for the formation of octahedral NiSe_2_/Ti_3_C_2_T_*x*_ hybrid through one-pot hydrothermal method

The brief synthetic procedure of Ti_3_C_2_T_*x*_ caped NiSe_2_ hybrid is shown in Scheme 1. The Ti_3_C_2_T_*x*_ MXene nanosheets (Figs. S1 and S2) were mixed within NiCl_2_/EDTA and Se/KOH solution, in which Ni(II) coordinated with EDTA first and formed a chelate complex, and thus could make a low concentration of free Ni(II) in the solution, which can prevent the deposition of NiSeO_3_ before the formation of selenides [51]. And then, the Ni(II) chelate complex could be adsorbed on the surface of Ti_3_C_2_T_*x*_ nanosheet due to the negatively charged terminal groups (–O or –OH). During the hydrothermal process, the electrostatic attraction between Ti_3_C_2_T_*x*_ and Ni(II) chelate complex ensures the Ti_3_C_2_T_*x*_ nanosheets wrapping on the surface of NiSe_2_ crystal, which results in the close contact between Ti_3_C_2_T_*x*_ and NiSe_2_, and thus could enhance the electrochemical performance of hybrid for both energy storage and conversion [7].

**Fig. 1:**
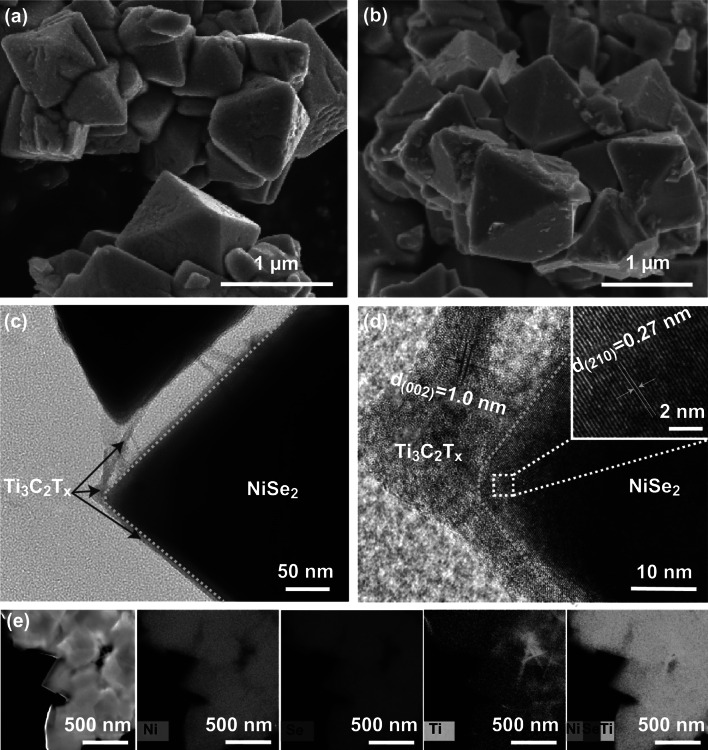
SEM images of NiSe_2_ without (**a**) and with Ti_3_C_2_T_*x*_ hybrid (**b**). **c**, **d** TEM image of NiSe_2_/Ti_3_C_2_T_*x*_ hybrid in different magnifications. **e** HAADF-STEM image of NiSe_2_/Ti_3_C_2_T_*x*_ hybrid and the corresponding EDX elemental mapping of Ni, Se, and Ti elements

The morphology of the as-prepared NiSe_2_ and NiSe_2_/Ti_3_C_2_T_*x*_ was characterized by SEM and TEM analyses. As shown in Fig. 1a, b, both unmodified NiSe_2_ and NiSe_2_/Ti_3_C_2_T_*x*_ exhibited a typical octahedral configuration under SEM, with the average sizes around 1 μm. However, the surface of the NiSe_2_/Ti_3_C_2_T_*x*_ crystal seemed relatively more glazing. Further analysis by TEM revealed that this phenomenon is possibly owing to the ultrathin layer of Ti_3_C_2_T_*x*_ nanosheet evenly covered on the surface of the octahedron-shaped NiSe_2_ particle (Fig. 1c), which might provide an additional electrical transport path for charge storage or electrocatalysis [53]. Moreover, the high-resolution TEM image in Fig. 1d demonstrates the single crystalline nature of the hybrid. The lattice fringe exhibits an interplanar distance of 0.27 nm, which is in accordance with the spacing of the (210) plane of in NiSe_2_ single crystal [27]. In addition, the lattice distance of 1.0 nm can be ascribed to the (002) facets of Ti_3_C_2_T_*x*_ nanosheet [38, 41]. It also need to be noted that there is transition phase between Ti_3_C_2_T_*x*_ nanosheet and NiSe_2_ single crystal, which cannot be well defined. Meanwhile, HAADF-STEM image and the corresponding EDX elemental mapping (Fig. 1e) show that distributions of Ni and Se in NiSe_2_/Ti_3_C_2_T_*x*_ hybrid were mostly homogeneous, whereas Ti exhibited the same distribution scale with less density. This also indicates that Ti_3_C_2_T_*x*_ nanosheet should be just evenly wrapped over the surface of NiSe_2_ crystal. In a word, these results confirmed that the hetero-nanostructure of NiSe_2_/Ti_3_C_2_T_*x*_ hybrid is comprised with crystallized NiSe_2_ octahedral crystal in the ultrathin Ti_3_C_2_T_*x*_ nanosheet cloak. As a conductive layer, the extra Ti_3_C_2_T_*x*_ nanosheet on the surface might be beneficial to strengthen the conductivity as well as the electrochemical activity of the original NiSe_2_ [7, 34].

**Fig. 2:**
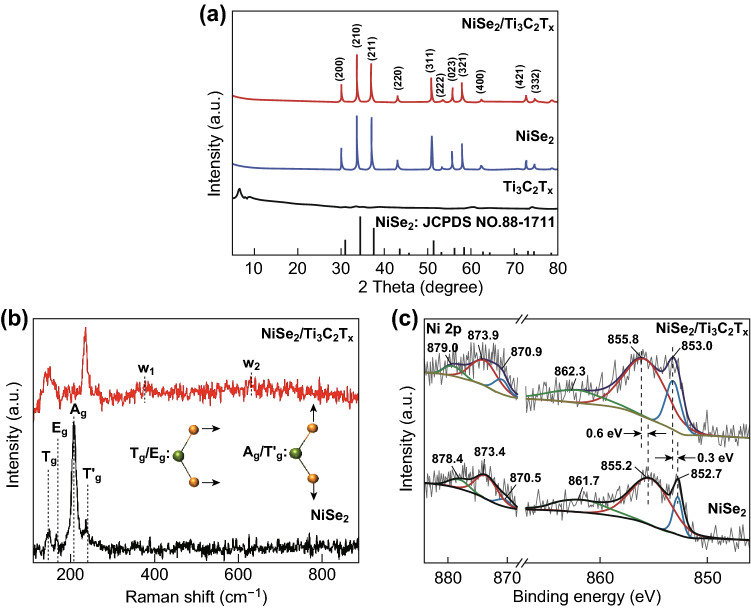
**a** XRD patterns of unmodified NiSe_2_ and NiSe_2_/Ti_3_C_2_T_*x*_ hybrid. **b** Comparison of Raman spectra for NiSe_2_ and NiSe_2_/Ti_3_C_2_T_*x*_ hybrid, and the inset is atom schematic of NiSe_2_. (The green ball is Ni atom, and the yellow ball is Se atom.) **c** Ni 2*p* XPS spectra of unmodified NiSe_2_ and NiSe_2_/Ti_3_C_2_T_*x*_ hybrid

The as-prepared NiSe_2_ and NiSe_2_/Ti_3_C_2_T_*x*_ samples were also investigated by XRD. As shown in Fig. 2a, the diffraction peaks of both materials at 29.9° (200), 33.6° (210), 36.9° (211), 42.9° (220), 50.7° (311), 53.2° (222), 55.5° (023), 57.8° (321), 62.2° (400), 72.6° (421), and 74.6° (332) are in well agreement with the pyrite NiSe_2_ (JCPDS NO. 88-1711), which echoes well with the results from high-resolution TEM, indicating the pyrite crystallized structure for NiSe_2_/Ti_3_C_2_T_*x*_ hybrid [20, 26]. Notably, the diffraction peak at 6.7° (002), corresponding to *c* lattice parameter of Ti_3_C_2_T_*x*_ sheet, was not observed in the XRD pattern of NiSe_2_/Ti_3_C_2_T_*x*_ hybrid, which is possibly because of the low content of Ti_3_C_2_T_*x*_ sheet in the hybrid [54]. Despite that, no obvious impurities were detected, indicating high purity and crystallinity for both crystals.

To further understand the interaction between Ti_3_C_2_T_*x*_ nanosheet and NiSe_2_ crystal in the composite, the Raman spectroscopy was employed. As displayed in Fig. 2b, for NiSe_2_/Ti_3_C_2_T_*x*_ hybrid, the four strong peaks (Tg, Eg, Ag, and Tg) at 149, 169, 210, and 240 cm^−1^ are in accordance with the Raman bands of pyrite NiSe_2_, which assigned to stretching and rotational modes of the Se–Se pairs in NiSe_2_ molecular, while the two weak peaks (*w*_1_ and *w*_2_) at 380 and 650 cm^−1^ are correlated with the Ti–C vibrations of Ti_3_C_2_T_*x*_ [30, 38]. Compared with the unmodified NiSe_2_, these four peaks of NiSe_2_/Ti_3_C_2_T_*x*_ hybrid obviously red-shift from the original coordinates of 154, 193, 237, and 269 cm^−1^, which is probably owing to the changing in the surface strain after the coating of Ti_3_C_2_T_*x*_ nanosheet, and also is indicative of a possible strong interfacial interaction between the NiSe_2_ and Ti_3_C_2_T_*x*_ in hybrid [7].

XPS was carried out to verify the interfacial interaction between NiSe_2_ and Ti_3_C_2_T_*x*_ (see Fig. S3 for the survey spectra). The Ni 2*p* spectrum has been fitted by considering two resolved doublets with a spin–orbit splitting around 18.0 eV between 2*p*_3/2_ and 2*p*_1/2_ and a fixed area ratio equal to 2:1 (Table S1). As shown in Fig. 2c, the Ni 2*p* spectrum of NiSe_2_/Ti_3_C_2_T_*x*_ hybrid exhibits peaks at binding energy of 853.0 and 870.9 eV, corresponding to the Ni 2*p*_3/2_ and Ni 2*p*_1/2_, respectively. The peaks at 855.8 and 873.9 eV are related to the oxidation state of Ni on the surface. Two satellite peaks at 862.3 and 879.0 eV were also observed [27, 55]. Compared with unmodified NiSe_2_, the characteristic peaks of Ni species from NiSe_2_/Ti_3_C_2_T_*x*_ hybrid are obviously shifted by 0.3 and 0.6 eV to higher binding energy. This obvious Ni 2*p* peak-shift toward higher binding energy confirms the strong interactions between NiSe_2_ and Ti_3_C_2_T_*x*_, which makes the Ni center in NiSe_2_ more positively charged and thus facilitates the electrostatic attraction of more anionic intermediates for fast redox process [56, 57]. This finding is in decent agreement with the Raman results and may be due to the Ti_3_C_2_T_*x*_ containing rich hydroxyl terminations with high electronegativity, which can strongly interact with NiSe_2_ [58]. Compared with traditional physical binding (Fig. S4), such binding method might be more efficient for redox process and charge transfer in NiSe_2_/Ti_3_C_2_T_*x*_ hybrid, consequently accelerating its electrochemical activities [7, 45].

### Supercapacitor Performance of NiSe_2_ and Ti_3_C_2_T_*x*_/NiSe_2_

**Fig. 3:**
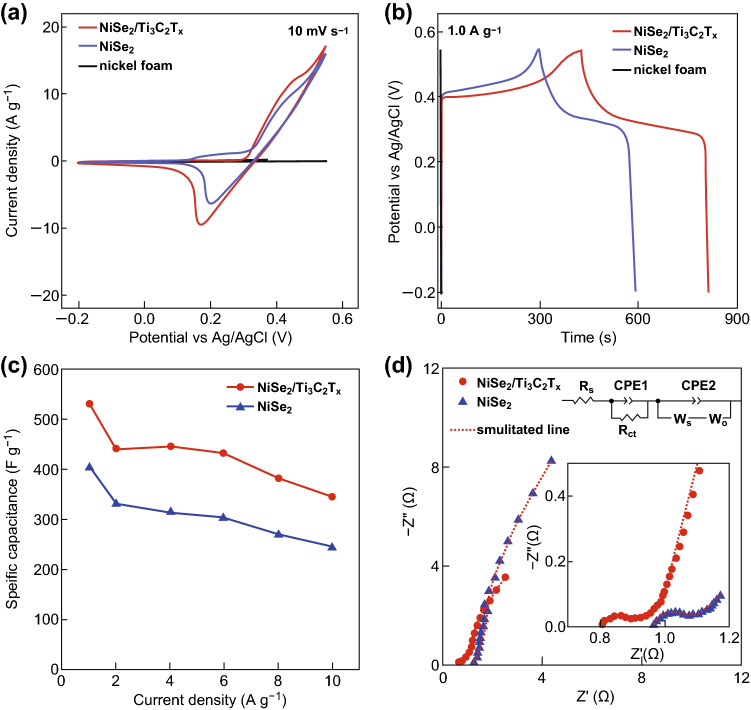
Supercapacitor performance in 2 M KOH solution: **a** CV profiles of unmodified NiSe_2_, NiSe_2_/Ti_3_C_2_T_*x*_, and bare nickel foam at 10 mV s^−1^. **b** GCD curves of unmodified NiSe_2_, NiSe_2_/Ti_3_C_2_T_*x*_, and bare nickel foam at 1.0 A g^−1^. **c** Variation of specific capacitances with current density for unmodified NiSe_2_ and NiSe_2_/Ti_3_C_2_T_*x*_. **d** Nyquist plots of unmodified NiSe_2_ and NiSe_2_/Ti_3_C_2_T_*x*_ in the range of 10 mHz to 100 kHz (inset is the high-frequency region and the equivalent circuit used to fit the experimental data)

To evaluate the electrochemical performance of NiSe_2_/Ti_3_C_2_T_*x*_ hybrid, the NiSe_2_/Ti_3_C_2_T_*x*_ hybrid materials and the unmodified NiSe_2_ were both applied as active materials for supercapacitor, and the electrochemical performance of nickel form substrate was also investigated. From the cyclic voltammetry (CV) curves, the bare nickel form substrate shows very small areas, indicating the low electrochemical activity. As demonstrated in Fig. 3a, the CV curves for unmodified NiSe_2_ and NiSe_2_/Ti_3_C_2_T_*x*_ hybrid exhibit a clear redox pair, which is attributed to the Faradic redox reaction and consistent well with previous reports, suggesting the pseudocapacitive characteristic of NiSe_2_ [25]. According to the literature, all divalent cations in NiSe_2_ were transformed into trivalent cations after positive sweep, and the reaction mechanism of charge storage may occur as shown in Eqs. 2 and 3 [24]:

2 $${\text{NiSe}}_{2} + {\text{H}}_{2} {\text{O}} + 1/2{\text{O}}_{2} \to {\text{Ni}}\left( {\text{OH}} \right)_{2} + 2{\text{Se}}$$

3 $${\text{Ni}}\left( {\text{OH}} \right)_{2} + {\text{OH}}^{ - } \leftrightarrow {\text{NiOOH}} + {\text{e}}^{ - }$$

Meanwhile, the paired redox peaks (see Fig. S5 for details) still existed with the increasing scan rate which indicates good ability of rapid oxidation reduction reaction for NiSe_2_ [15]. However, with the increase in the scan rate, an asymmetry of redox peaks was detected, which may be due to the polarization effect as well as ohmic resistance during the Faradic process [59, 60]. In addition, the symmetrical time of charge and discharge in all cases (see Fig. S6 for details) indicates high coulombic efficiencies of as-prepared electrodes and in good agreement with the CV curves. In particular, NiSe_2_/Ti_3_C_2_T_*x*_ hybrid exhibits much larger CV area and longer charge–discharge time (Fig. 3b) compared with the unmodified NiSe_2_, verifying the higher supercapacitor performance. From the Galvanostatic charging/discharging curves (GCD) curves, the highest specific capacitance of NiSe_2_/Ti_3_C_2_T_*x*_ was estimated to be 531.2 F g^−1^ at 1 A g^−1^, which is 31% larger than that of the unmodified NiSe_2_ at same current densities. Moreover, a specific capacitance of 348 F g^−1^ for NiSe_2_/Ti_3_C_2_T_*x*_ hybrid could still be achieved at a higher current density of 10 A g^−1^, exhibiting a relatively good rate capability with the retained capacitance of 66%, which is 8% larger than that of the unmodified NiSe_2_. This considerably enhanced electrochemical performance and improved rate capability of the NiSe_2_/Ti_3_C_2_T_*x*_ hybrid which might be caused by the interfacial effect from the interfacial interaction of Ti_3_C_2_T_*x*_, which improves the electrochemical activities of the materials.

To further investigate the inner electrochemical mechanism of the performance facilitating process, the EIS was carried out for both NiSe_2_/Ti_3_C_2_T_*x*_ hybrid and unmodified NiSe_2_. As shown in Fig. 3d, the Nyquist plots for both materials exhibit the similar pattern with two semicircles in the high-frequency region and a linear line in the low frequency area. The first and second semicircles are related to the charge-transfer process and electrolyte infiltration process on the surface, respectively, whereas the linear part is related to finite Nernst diffusion in the surface and semi-infinite Warburg diffusion process in the bulk [12]. In Fig. 3d, a simulated equivalent circuit is introduced to fit the Nyquist plots, where the *R*_s_ means solution resistance; CPE1 and CPE2 are constant phase element; *R*_ct_ represents the charge-transfer resistance; *W*_s_ and *W*_o_ are the finite Nernst diffusion impedance and semi-infinite Warburg diffusion impedance, respectively [12]. The detailed fitting parameters are included in Table S2. The results manifest a charge-transfer resistance of 95.4 mΩ for NiSe_2_/Ti_3_C_2_T_*x*_ hybrid, which is obviously lower than that of unmodified NiSe_2_ (127.4 mΩ). This reduced charge-transfer resistance for NiSe_2_/Ti_3_C_2_T_*x*_ hybrid should be attributed to the enhanced conductivity from the chemical combination of high conductive Ti_3_C_2_T_*x*_ sheet. Meanwhile, the Warburg diffusion resistance of the NiSe_2_/Ti_3_C_2_T_*x*_ hybrid is 31.6% smaller than that of unmodified NiSe_2_, indicating a higher ion diffusion rate. The low diffusion resistance of ion transport should be owing to the two-dimensional morphology and the extra redox active sites from terminal group on the Ti_3_C_2_T_*x*_ sheet surface [40, 44]. In sum, the EIS spectrum analysis demonstrates that the introduction of Ti_3_C_2_T_*x*_ provides lowered charge-transfer resistance as well as enhanced ion diffusion rate, therefore facilitating a higher specific capacitance for NiSe_2_/Ti_3_C_2_T_*x*_ hybrid [7].

In addition, the long-term stability of the two samples also has been tested via the GCD method at 4 A g^−1^ (Fig. S7). It showed that the capacitance retention of NiSe_2_/Ti_3_C_2_T_*x*_ hybrid is 47.3% larger than that of unmodified NiSe_2_ after 1000 cycles, suggesting that the Ti_3_C_2_T_*x*_ sheet can also improve the stability of NiSe_2_ during electrochemical process. This stabilization effect might be attributed to two aspects. Firstly, since the NiSe_2_ is known to form irreversible oxide counterparts in the electrochemical cycling, the Ti_3_C_2_T_*x*_ sheet can serve as a protective layer from its oxidation [20, 31]. Secondly, similar to the modification on rGO and other 2D carbonaceous materials [61, 62], the interfacial interaction of Ti_3_C_2_T_*x*_ sheet should favor the defective sites on NiSe_2_ particle surface, which, to a large extent, amends the deficiency of the original NiSe_2_ nanocrystal. However, these deductions still need further verification in the future.

### HER Performance of NiSe_2_ and NiSe_2_/Ti_3_C_2_T_*x*_

**Fig. 4:**
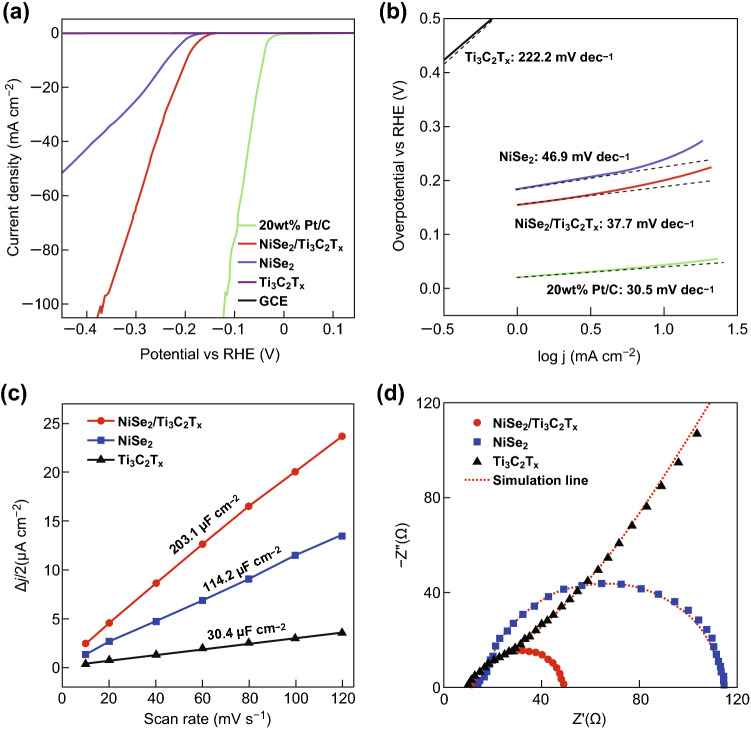
HER catalytic performance in 0.5 M H_2_SO_4_ solution: **a** LSV curves of unmodified NiSe_2_, NiSe_2_/Ti_3_C_2_T_*x*_ hybrid, pure Ti_3_C_2_T_*x*_, 20 wt%Pt/C, and glassy carbon electrode at the scan rate of 5 mV s^−1^. **b** Tafel plots of unmodified NiSe_2_, NiSe_2_/Ti_3_C_2_T_*x*_ hybrid, pure Ti_3_C_2_T_*x*_, and 20 wt%Pt/C. **c** The extracted double-layer capacitances of different electrodes using a cyclic voltammetry method. **d** EIS Nyquist plots in the range of 10 mHz to 100 kHz

To estimate its potential multifunction, NiSe_2_/Ti_3_C_2_T_*x*_ hybrid was employed as the catalytic materials in hydrogen evolution reaction, where the catalytic materials would decrease the reaction barrier. For comparison, HER activity measurements were also performed for the pure Ti_3_C_2_T_*x*_, unmodified NiSe_2_, 20%Pt/C, and glassy carbon under the same conditions. From LSV curves in Fig. 4a, as expected, the 20% Pt/C displays remarkable HER activity (10 mA cm^−2^ at 44 mV vs. RHE), while pure Ti_3_C_2_T_*x*_ and glassy carbon display no obvious catalytic activity for H_2_ evolution. In contrasted, the NiSe_2_/Ti_3_C_2_T_*x*_ exhibits an enormous increase in the cathodic current density compared with the NiSe_2_ and the Ti_3_C_2_T_*x*_, exhibiting an overpotential of 200.0 mV at a current density of 10 mA cm^−2^, which is lower than unmodified NiSe_2_ of 239.0 mV under the same current density. Moreover, the overpotential required for the NiSe_2_/Ti_3_C_2_T_*x*_ hybrid and unmodified NiSe_2_ to produce a current density of 45 mA cm^−2^ is 269.0 and 418.1 mV, respectively. It is obvious that the NiSe_2_/Ti_3_C_2_T_*x*_ hybrid possesses a better HER catalytic activity than unmodified NiSe_2_. The enhanced performance of the NiSe_2_/Ti_3_C_2_T_*x*_ electrode can be attributed to the charge transfer from NiSe_2_ to Ti_3_C_2_T_*x*_, which may provide a faster adsorption kinetics and higher utilization of active sites for high HER efficiency [14].

In order to demonstrate the kinetics of the electrodes during the HER process, the Tafel analysis was carried out on the polarization curve. As shown in Fig. 4b, the NiSe_2_/Ti_3_C_2_T_*x*_ hybrid with low Tafel slope of 37.7 mV dec^−1^ is nearly comparable to that of Pt catalyst (30.5 mV dec^−1^) and smaller than that of the unmodified NiSe_2_ (46.9 mV dec^−1^) as well as unmodified Ti_3_C_2_T_*x*_ (222.2 mV dec^−1^), which implies that the electrocatalytic reaction follows the Volmer–Heyrovsky pathway for the NiSe_2_/Ti_3_C_2_T_*x*_ hybrid [7]. In addition, the exchange current density (*j*_0_) at the thermodynamic redox potential (*η *= 0), another key factor to evaluate catalytic property, can be extracted by extrapolating the Tafel plots to the *x*-axis [30]. The *j*_0_ value (147.5 μA cm^−2^) for the NiSe_2_/Ti_3_C_2_T_*x*_ hybrid is higher than that of the unmodified NiSe_2_ (56.8 μA cm^−2^), indicating a better catalytic activity of the NiSe_2_/Ti_3_C_2_T_*x*_ hybrid (Table S3). These results clearly demonstrate an enhanced catalytic performance of NiSe_2_/Ti_3_C_2_T_*x*_ hybrid, which is possibly owing to the interfacial interaction between the Ti_3_C_2_T_*x*_ sheet and NiSe_2_ crystal, inducing the charge-transfer process. As shown in Figs. 4c and S8, the double-layer capacitance *C*_dl_ for NiSe_2_/Ti_3_C_2_T_*x*_ hybrid (203.1 μF cm^−2^) also exhibits large improvement compared with unmodified NiSe_2_ (114.2 μF cm^−2^), which suggests that the Ti_3_C_2_T_*x*_ sheet wrapping on NiSe_2_ surface not only facilitates changer transfer, but also improves the utilization of active site as well [32].

The EIS measurements in 0.5 M H_2_SO_4_ electrolyte provide further details on the enhanced HER process. As shown in Fig. 4d, the Nyquist plots are fitted by equivalent circuit model (Fig. S9) [63], and the simulated parameters are summarized in Table S2. The NiSe_2_/Ti_3_C_2_T_*x*_ hybrid exhibits the lower *R*_ct_ (33.9 Ω) than that of unmodified NiSe_2_ (95.7 Ω), indicating a higher electrochemical reaction rate and more efficient charge-transfer process for hybrid [64]. This enhanced conductivity could result from the chemical coupling with Ti_3_C_2_T_*x*_ sheet, a better conductor. Consequently, the high conductivity of the NiSe_2_/Ti_3_C_2_T_*x*_ hybrid guarantees the higher apparent catalytic activity toward HER since less potential is needed for driving the current transport across the catalyst [48]. In addition, the long-term stability test revealed a superior durability of the NiSe_2_/Ti_3_C_2_T_*x*_ hybrid (Fig. S10), where the NiSe_2_/Ti_3_C_2_T_*x*_ hybrid showed negligible degradation in the current density after 2000 cycles, which is also positively related to the combination of Ti_3_C_2_T_*x*_ sheet [33]. To demonstrate further the good stability, time-dependent current density curve for NiSe_2_/Ti_3_C_2_T_*x*_ under constant overpotential of − 0.25 V versus RHE was also conducted for 10 h. As shown in Fig. S11, the current density of the NiSe_2_/Ti_3_C_2_T_*x*_ hybrid has slight decrease compared with pure NiSe_2_.

In general, certain requirements such as good electrical conductivity, robust structure, large active surface area, and fast diffusion pathway, are applicable to achieve high-performance materials for energy storage and electrocatalysis [48]. The unique structural superiorities make NiSe_2_/Ti_3_C_2_T_*x*_ hybrid a well-fit candidate for these demands. Firstly, the NiSe_2_ are stabilized by Ti_3_C_2_T_*x*_ MXene sheet wrapping on the surface, and their outstanding electrical properties are secured. Moreover, the Ti_3_C_2_T_*x*_ MXene sheet significantly promotes the electronic coupling by acting as the 2D conductive linker for fast charge transfer. Finally, the functional terminal group on Ti_3_C_2_T_*x*_ MXene surface provides the possibility for chemical coupling with NiSe_2_ crystal, which enables the strong interfacial interaction for fast charge transfer and high stability against repeated electrochemical cycling as well. These advantages for the synergism of NiSe_2_ crystal and Ti_3_C_2_T_*x*_ MXene sheet have led to a remarkable improvement in electrochemical activity and durability for supercapacitor and HER. Furthermore, compared with recently reported supercapacitor materials and HER catalysts (Table S4 and Table S5, respectively), the NiSe_2_/Ti_3_C_2_T_*x*_ hybrid has shown superior performance, verifying an enormous potential in multifunctional applications.

## Conclusions

In summary, a novel NiSe_2_/Ti_3_C_2_T_*x*_ hybrid has been successfully synthesized, by wrapping NiSe_2_ octahedral crystal with ultrathin Ti_3_C_2_T_*x*_ MXene nanosheet via a one-pot hydrothermal method. Its composition and morphology have been analyzed through varies techniques, establishing a strong interfacial interaction between NiSe_2_ octahedral crystal and the evenly distributed ultrathin Ti_3_C_2_T_*x*_ MXene nanosheet layer on its surface. A high specific capacitance of 531.2 F g^−1^ at 1 A g^−1^ for supercapacitor, low overpotential of 200 mV at 10 mA g^−1^, and small Tafel slope of 37.7 mV dec^−1^ for HER are achieved by employing NiSe_2_/Ti_3_C_2_T_*x*_ hybrid as the electrode material. Furthermore, greater cycling stabilities for NiSe_2_/Ti_3_C_2_T_*x*_ hybrid in both supercapacitor and HER have also been achieved. These significant improvements compared with unmodified NiSe_2_ should be owing to the strong interfacial interaction between NiSe_2_ octahedral crystal and Ti_3_C_2_T_*x*_ MXene which provides enhanced conductivity, faster charge transfer as well as more abundant active sites and also highlight the promising potentials in combinations of MXene with metal selenides for multifunctional applications such as energy storage and conversion.

## Electronic supplementary material

Below is the link to the electronic supplementary material.

Supplementary material 1 (PDF 712 kb)
