# Supplementary material for: Ultrathin Ti3C2Tx (MXene) Nanosheet-Wrapped NiSe2 Octahedral Crystal for Enhanced Supercapacitor Performance and Synergetic Electrocatalytic Water Splitting
Source: Nanomicro Lett. 2019 Apr 4;11:31. doi: 10.1007/s40820-019-0261-5 (PMC7770682; doi:10.1007/s40820-019-0261-5)
Supplement: Supplementary file 1 — Supplementary material 1 (PDF 712 kb) [file 40820_2019_261_MOESM1_ESM.pdf]

Supporting Information for

Ultrathin $Ti_3C_2T_x$ (MXene) Nanosheets Wrapped $NiSe_2$ Octahedral Crystal for Enhanced Supercapacitor Performance and Synergetic Electrocatalytic Water Splitting

Hanmei Jiang[1, 2, †], Zegao Wang[1, 3, †], Qian Yang[4], Luxi Tan[2], Lichun Dong[2, *], Mingdong Dong[1, *]

[1]Interdisciplinary Nanoscience Center (iNANO), Aarhus University, Aarhus-C 8000, Denmark

[2]School of Chemistry and Chemical Engineering, Key Laboratory of Low-grade Energy Utilization Technologies & Systems of the Ministry of Education, Chongqing University, Chongqing 400044, People's Republic of China

[3]College of Materials Science and Engineering, Sichuan University, Chengdu 610065, People's Republic of China

[4]College of Chemistry and Molecular Engineering, Peking University, Beijing 100871, People's Republic of China

†Hanmei Jiang and Zegao Wang contributed equally to this work

*Corresponding authors. E-mail: lcdong72@cqu.edu.cn (Lichun Dong); dong@inano.au.dk (Mingdong Dong)

S1 The Morphology and Characteration of As-Prepared $Ti_3C_2T_x$ and $NiSe_2/Ti_3C_2T_x$

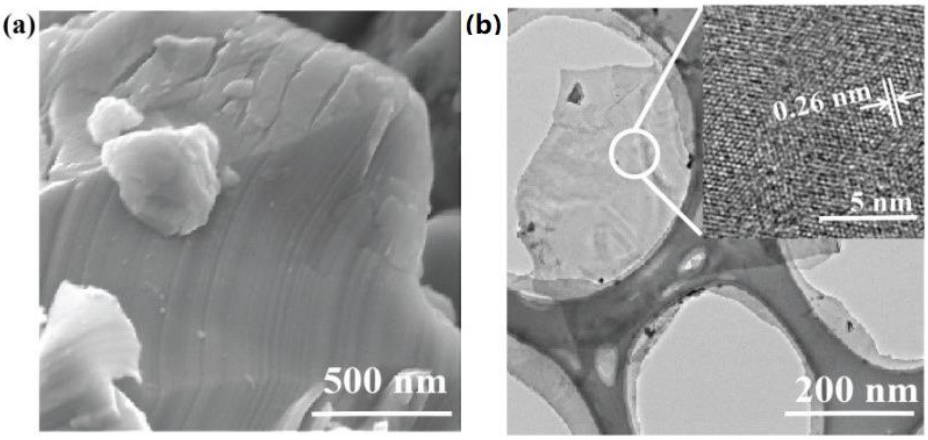

Fig. S1 a SEM image for commercial Ti_3AlC_2; **b** TEM for $Ti_3C_2T_x$ sheets (inset is HRTEM for $Ti_3C_2T_x$ sheets, the equiangular lattice spacing about 0.26 nm is a typical (100) planes of $Ti_3C_2T_x$) [S1]

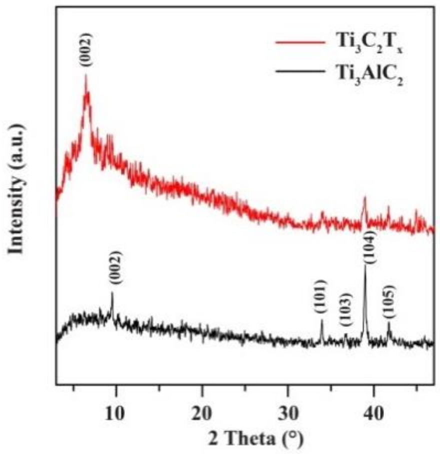

Fig. S2 XRD patterns of commercial Ti_3AlC_2 crystals and as-prepared $Ti_3C_2T_x$ crystals

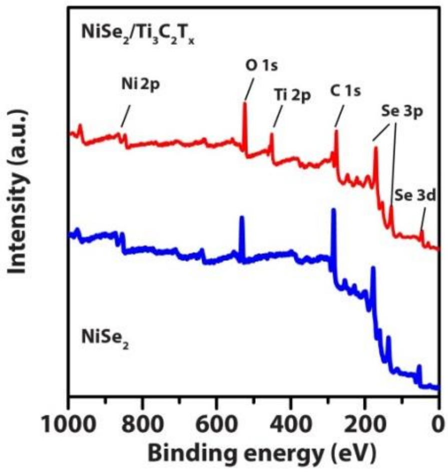

Fig. S3 XPS survey spectrum of the $NiSe_2/Ti_3C_2T_x$ hybrid, and C 1s (284.8eV) was used to calibrate all the XPS peaks before comparison

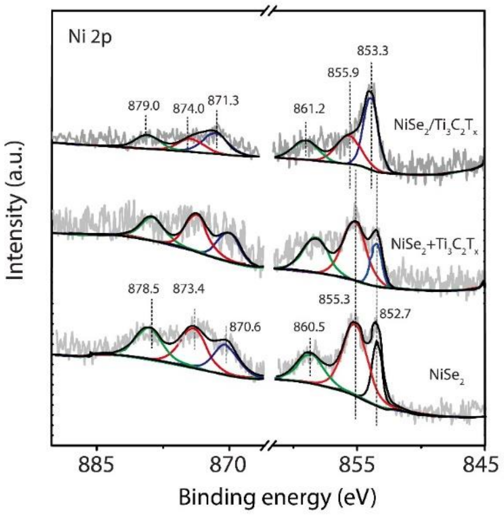

Fig. S4 Ni 2p XPS spectra of unmodified $NiSe_2$, $NiSe_2+Ti_3C_2T_x$ physical mixture, and $NiSe_2/Ti_3C_2T_x$ hybrid

Table S1a Fitting parameters of Ni 2p XPS spectra for the unmodified $NiSe_2$

	bare $NiSe_2$					
	**Ni $2p_{3/2}$**	**Ni $2p_{1/2}$**	**$Ni_{oxidation}$ $2p_{3/2}$**	**$Ni_{oxidation}$ $2p_{1/2}$**	Satellite **$2p_{3/2}$**	satellite **$2p_{1/2}$**
**Binding energy (eV)**	852.7	870.5	855.2	873.4	861.7	878.4
**Area**	646.7	323.4	3441.0	1720.5	1278.6	639.3
**FWHM (eV)**	1.0	2.8	4.4	4.4	5.9	3.9
**Concentrate (%)**	8.1	4.1	36.7	18.3	21.9	10.9

Table S1b Fitting parameters of Ni 2p XPS spectra for $NiSe_2/Ti_3C_2T_x$ hybrid

	$NiSe_2/Ti_3C_2T_x$					
	**Ni $2p_{3/2}$**	**Ni $2p_{1/2}$**	**$Ni_{oxidation}$ $2p_{3/2}$**	**$Ni_{oxidation}$ $2p_{1/2}$**	Satellite **$2p_{3/2}$**	satellite **$2p_{1/2}$**
**Binding energy (eV)**	853.0	870.9	855.8	873.9	862.3	879.0
**Area**	870.8	435.4	2910.8	1455.4	815.3	407.7
**FWHM (eV)**	1.6	2.8	4.6	5.2	6.0	3.4
**Concentrate (%)**	12.6	6.3	42.2	21.1	11.8	5.9

The CasaXPS softerware was used to fit the XPS spectra. The Ni 2p spectrum had been fitted by considering two resolved doublets with a spin–orbit splitting around 18.0 eV between $2p_{3/2}$ and $2p_{1/2}$ and a fixed area ratio about equal to 2:1.

S2 Addition Information of Supercapacitor Performance

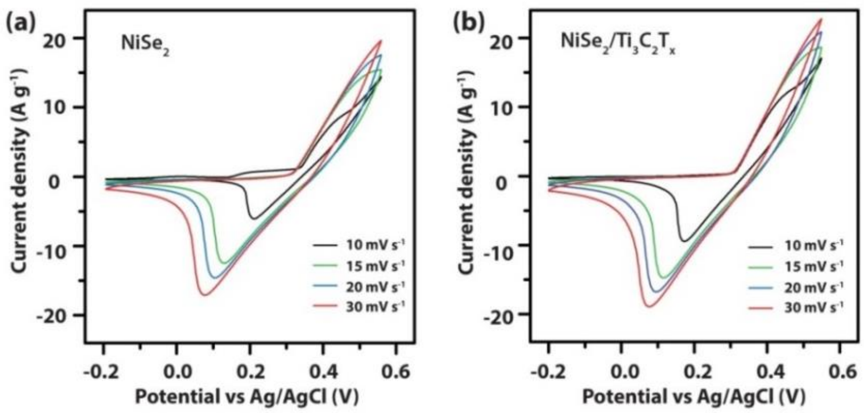

Fig. S5 CV curves at different scan rates from 10 to 30 mV s^{-1} toward **a** unmodified $NiSe_2$ and **b** $NiSe_2/Ti_3C_2T_x$hybrid

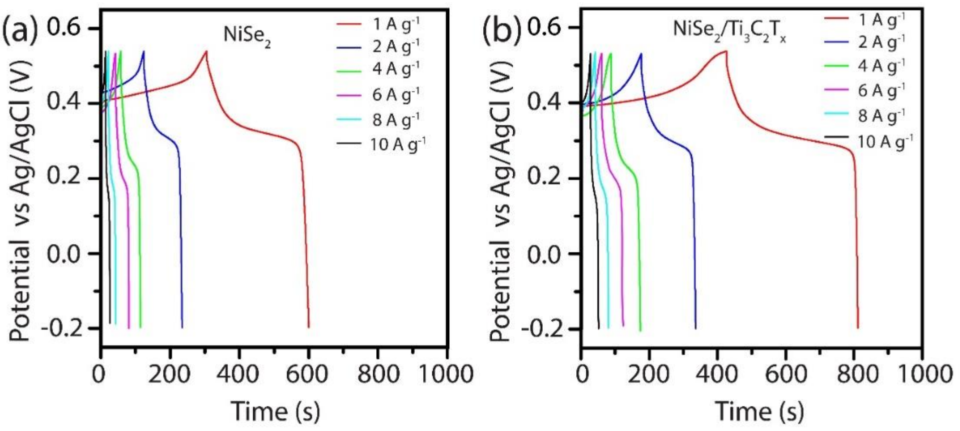

Fig. S6 GCD curves at different current densities from 1 to10 A g^{-1} toward **a** unmodified $NiSe_2$ and **b** $NiSe_2/Ti_3C_2T_x$hybrid

Table S2 The simulatedparameters of the unmodified$NiSe_2$ and $NiSe_2/Ti_3C_2T_x$ hybrid electrodes impedance spectra according to the equivalent circuit for supercapacitor

Sample	R_s (mΩ)	CPE1 (mF)	CPE2 (mF)	R_{ct} (mΩ)	W_s (mΩ $s^{-0.5}$)	W_o (mΩ $s^{-0.5}$)
$NiSe_2$	96.3	43.1	38.3	127.4	166.5	7.9
$NiSe_2/Ti_3C_2T_x$	79.9	58.0	91.0	95.4	92.7	5.4

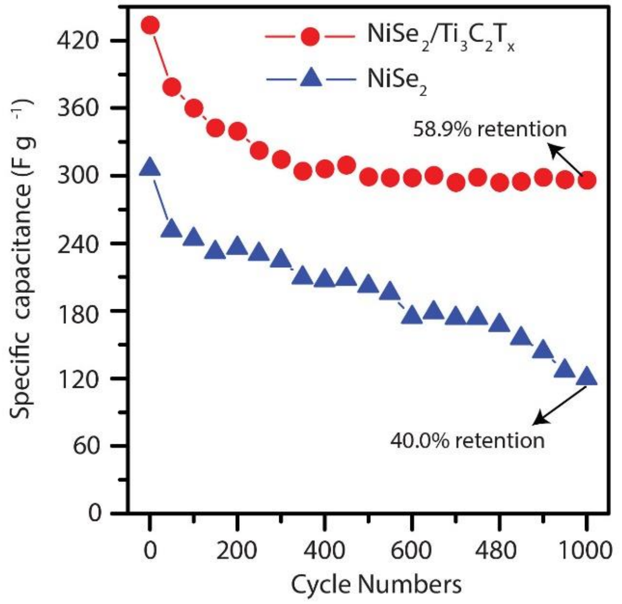

Fig. S7 Cyclic performance of $NiSe_2/Ti_3C_2T_x$ hybrid and unmodified $NiSe_2$ for 1000 cycles at a current density of 4 A g^{-1}

S3 Addition Information of HER

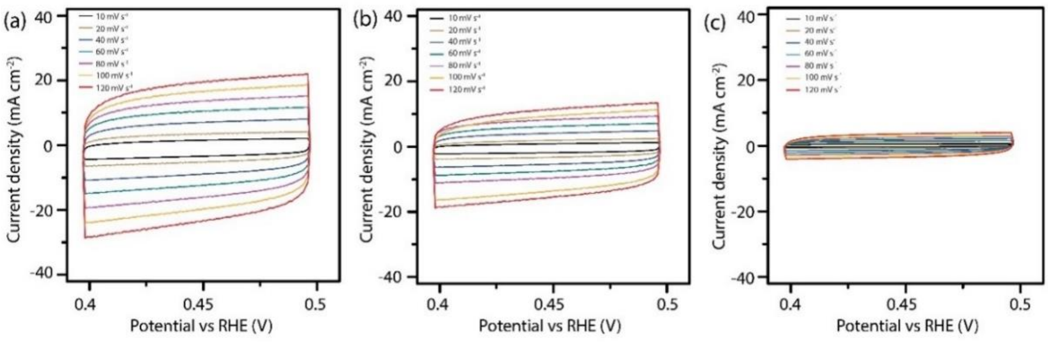

Fig. S8 CV curves of **a** $NiSe_2/Ti_3C_2T_x$ hybrid, **b** unmodified $NiSe_2$, and **c** $Ti_3C_2T_x$ are taken in a selected potential rangewithout faradaic current at different potential scanning rates range from 10 to 120 mV s^{-1}

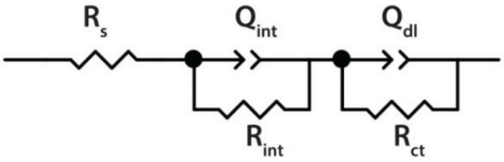

Fig. S9 Electrical equivalent circuit used to model the system of the $NiSe_2/Ti_3C_2T_x$ hybrid, unmodified $NiSe_2$, and $Ti_3C_2T_x$ investigated with EIS, in which R_s is solution resistance, R_{int} means the electrode-electrolyte interface resistance, R_{ct} is the charge transfer resistance , Q_{int} and Q_{dl} are constant phase elements [S2]

Table S3 Addition HER parameters and simulated parameters of the electrodes comprised of $NiSe_2/Ti_3C_2T_x$hybrid, unmodified$NiSe_2$ and $Ti_3C_2T_x$ according to the equivalent circuit

Samples	Overpotential (mV)/Current Density(mA cm^{-2})	Exchange current density (μA cm^{-2})	R_s (Ω)	R_{int} (Ω)	R_{ct} (Ω)	Q_{int} (mF)	Q_{ct} (mF)
$NiSe_2/Ti_3C_2T_x$	269.0/45	147.5	10.7	4.2	33.9	0.4	0.06
$NiSe_2$	418.1/45	56.8	14.8	9.3	95.7	0.9	0.02
$Ti_3C_2T_x$	/	/	8.4	/	11.2	0.1	0.6

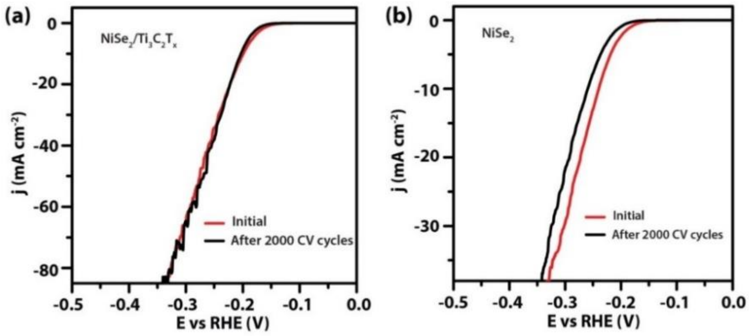

Fig. S10 Polarization curves of **a** $NiSe_2/Ti_3C_2T_x$ hybrid and **b** unmodified $NiSe_2$ before and after 2000 CV cycles at 100 mV s^{-1} for stability test

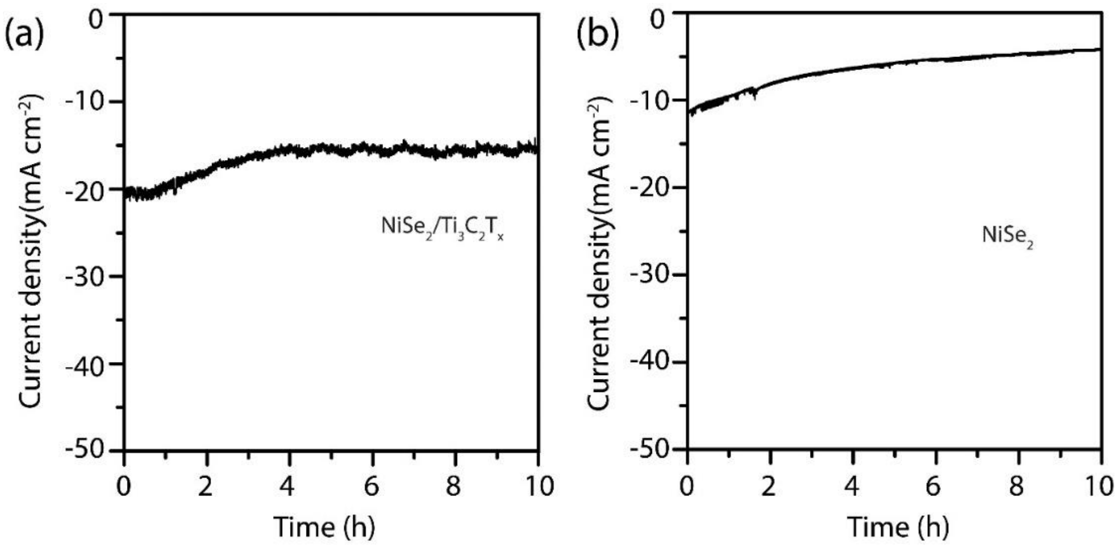

Fig. S11 Time-dependent currentdensity curve of **a** $NiSe_2/Ti_3C_2T_x$ hybrid and **b** unmodified $NiSe_2$ under static potential of -0.25V *vs* RHE for 10 h

S4 Supercapacitor and HER Performance of Other Masteries in the Literatures

Table S4 The comparison on the supercapacitor performance of our hybrid with other transition metal selenides and MXene-based composites in the literatures

Samples	Electrode	Electrolyte	Loading mass (mg cm^{-2})	Cs (F g^{-1})	References
$NiSe_2/Ti_3C_2T_x$	Nickel foam	2M KOH	8.0±0.2	532.1 at 1 A g^{-1}	This work
$NiSe_2$	Nickel foam	2M KOH	8.0±0.2	405.6 at 1 A g^{-1}	This work
$NiSe_2$	Nickel foam	2M KOH	10.0	341 at 1 A g^{-1}	[S3]
NiSe	Nickel foam	2M KOH	23.4	298 at 1 A g^{-1}	[S4]
$NiSe_2$	Graphite	1M KOH	0.5	75 at 1 mA cm^{-2}	[S5]
$CoSe_2$/CC	Carbon cloth	1M KOH	0.53	713.2 at 1 mA cm^{-2}	[S6]
$Co_{0.85}Se$	Nickel foam	2M KOH	5.0	280 at 1 A g^{-1}	[S7]
$FeSe_2$	Nickel foam	2M KOH	/	171 at 1 A g^{-1}	[S8]
$MoO_3/Ti_3C_2T_x$	Nickel foam	1M KOH	/	94 at 1 A g^{-1}	[S9]
$MnO_2/Ti_3C_2T_x$	Nickel foam	1M $NaSO_4$	4	212.1 at 1 A g^{-1}	[S10]

Table S5 Summary of electrochemical parameters of some catalyst in previous works and our work for HER

Samples	Tafel slope (mV dec^{-1})	Overpotential (mV)/Current Density (mA cm^{-2})	Exchange current density (μA cm^{-2})	Electrolyte	References
$NiSe_2/Ti_3C_2T_x$	37.7	200/10	147.5	0.5M H_2SO_4	This work
$NiSe_2$	46.9	239/10	56.8	0.5M H_2SO_4	This work
$NiSe_2$	29.4	200/10	/	0.5M H_2SO_4	[S11]
NiSe/NF	59.8	224/10	15.7	0.5M H_2SO_4	[S12]
$NiSe_2$ NCs	44	205/10	2	0.5M H_2SO_4	[S13]
$NiSe_2$/CNWs	38.7	225	/	0.5M H_2SO_4	[S14]
NiS_2	48.8	/	0.02	0.5M H_2SO_4	[S15]
CoS_2	51.6	210/10	15.1	0.5M H_2SO_4	[S16]
$CoO/CoSe_2$	131	337/10	33.2	0.5M H_2SO_4	[S17]
MoS_2	50	280/10	/	0.5M H_2SO_4	[S18]
$MoS_2/Ti_3C_2T_x$	74	332/10	/	0.5M H_2SO_4	[S19]
Ni_2P	48	220/10	/	0.5M H_2SO_4	[S20]
CoP	104.8	250/10	63	0.5M H_2SO_4	[S21]
FeP	67	249/10	/	0.5M H_2SO_4	[S22]

"/": not provided in the data

Supplementary References

[S1]M. Ghidiu, M.R. Lukatskaya, M.Q. Zhao, Y. Gogotsi, M.W. Barsoum, Conductive two-dimensional titanium carbide 'clay' with high volumetric capacitance. Nature **516**(7529), 78-81 (2014). http://doi:10.1038/nature13970

[S2]H.A. Bandal, A.R. Jadhav, A.H. Tamboli, H. Kim, Bimetallic iron cobalt oxide self-supported on Ni-Foam: An efficient bifunctional electrocatalyst for oxygen and hydrogen evolution reaction. Electrochim. Acta **249,** 253-262 (2017). http://doi:10.1016/j.electacta.2017.07.178

[S3]M. Lu, X.-P. Yuan, X.-H. Guan, G.-S. Wang, Synthesis of nickel chalcogenide hollow spheres using an l-cysteine-assisted hydrothermal process for efficient supercapacitor electrodes. J. Mater. Chem. A **5**(7), 3621-3627 (2017). http://doi:10.1039/c6ta10426f

[S4]K. Guo, F. Yang, S. Cui, W. Chen, L. Mi, Controlled synthesis of 3D hierarchical NiSe microspheres for high-performance supercapacitor design. RSC Adv. **6**(52), 46523-46530 (2016). http://doi:10.1039/c6ra06909f

[S5]N.S. Arul, J.I. Han, Facile hydrothermal synthesis of hexapod-like two dimensional dichalcogenide $NiSe_2$ for supercapacitor. Mater. Lett. **181,** 345-349 (2016). http://doi:10.1016/j.matlet.2016.06.065

[S6]T. Chen, S. Li, J. Wen, P. Gui, G. Fang, Metal-organic framework template derived porous $CoSe_2$ nanosheet arrays for energy conversion and storage. ACS Appl. Mater. Interfaces **9**(41), 35927-35935 (2017). http://doi:10.1021/acsami.7b12403

[S7]H. Peng, G. Ma, K. Sun, Z. Zhang, J. Li, X. Zhou, Z. Lei, A novel aqueous asymmetric supercapacitor based on petal-like cobalt selenide nanosheets and nitrogen-doped porous carbon networks electrodes. J. Power Source **297,** 351-358(2015). http://doi:https://doi.org/10.1016/j.jpowsour.2015.08.025

[S8]C. Ji, F. Liu, L. Xu, S. Yang, Urchin-like $NiCo_2O_4$ hollow microspheres and $FeSe_2$ micro-snowflakes for flexible solid-state asymmetric supercapacitors. J. Mater. Chem. A **5**(11), 5568-5576 (2017). http://doi:10.1039/c6ta11001k

[S9]J. Zhu, X. Lu, L. Wang, Synthesis of a $MoO_3/Ti_3C_2T_x$ composite with enhanced capacitive performance for supercapacitors. RSC Adv. **6**(100), 98506-98513 (2016). http://doi:10.1039/c6ra15651g

[S10] R.B. Rakhi, B. Ahmed, D. Anjum, H.N. Alshareef, Direct chemical synthesis of MnO_2 nanowhiskers on transition-metal carbide surfaces for supercapacitor applications. ACS Appl. Mater. Interfaces **8**(29), 18806-18814 (2016). http://doi:10.1021/acsami.6b04481

[S11] B. Yu, X. Wang, F. Qi, B. Zheng, J. He, J. Lin, W. Zhang, Y. Li, Y. Chen, Self-assembled coral-like hierarchical architecture constructed by $NiSe_2$ nanocrystals with comparable hydrogen-evolution performance of precious platinum catalyst. ACS Appl. Mater. Interfaces **9**(8), 7154-7159 (2017). http://doi:10.1021/acsami.6b15719

[S12] H.Q. Zhou, Y.M. Wang, R. He, F. Yu, J.Y. Sun, F. Wang, Y.C. Lan, Z.F. Ren, S. Chen, One-step synthesis of self-supported porous $NiSe_2$/Ni hybrid foam: An efficient 3D electrode for hydrogen evolution reaction. Nano Energy **20,** 29-36 (2016). http://doi:10.1016/j.nanoen.2015.12.008

[S13] I.H. Kwak, H.S. Im, D.M. Jang, Y.W. Kim, K. Park, Y.R. Lim, E.H. Cha, J. Park, $CoSe_2$ and $NiSe_2$ nanocrystals as superior bifunctional catalysts for electrochemical and photoelectrochemical water splitting. ACS Appl. Mater. Interfaces **8**(8), 5327-5334 (2016). http://doi:10.1021/acsami.5b12093

[S14] D.Y. Song, H.Q. Wang, X.Q. Wang, B. Yu, Y.F. Chen, $NiSe_2$ nanoparticles embedded in carbon nanowires as highly efficient and stable electrocatalyst for hydrogen evolution reaction. Electrochim. Acta **254**, 230-237 (2017). http://doi:10.1016/j.electacta.2017.09.056

[S15] M.S. Faber, M.A. Lukowski, Q. Ding, N.S. Kaiser, S. Jin, Earth-abundant metal pyrites (FeS_2, CoS_2, NiS_2, and Their Alloys) for highly efficient hydrogen evolution and polysulfide reduction electrocatalysis. J. Phy. Chem. C **118**(37), 21347-21356 (2014).

http://doi:10.1021/jp506288w

[S16] M.S. Faber, R. Dziedzic, M.A. Lukowski, N.S. Kaiser, Q. Ding, S. Jin, High-performance electrocatalysis using metallic cobalt pyrite (CoS_2) micro and nanostructures. J. Am. Chem. Soc. **136**(28), 10053-10061 (2014). http://doi:10.1021/ja504099w

[S17] K. Li, J. Zhang, R. Wu, Y. Yu, B. Zhang, Anchoring CoO domains on $CoSe_2$ nanobelts as bifunctional electrocatalysts for overall water splitting in neutral media. Adv.Sci. **3**(6), 1500426 (2016). http://doi:doi:10.1002/advs.201500426

[S18] Y. Yang, H.L. Fei, G.D. Ruan, C.S. Xiang, J.M. Tour, Edge-oriented MoS_2 nanoporous films as flexible electrodes for hydrogen evolution reactions and supercapacitor devices. Adv. Mater. **26**(48), 8163-8168 (2014). http://doi:10.1002/adma.201402847

[S19] X. Wu, Z. Wang, M. Yu, L. Xiu, J. Qiu, Stabilizing the MXenes by carbon nanoplating for developing hierarchical nanohybrids with efficient lithium storage and hydrogen evolution capability. Adv. Mater. **29**(24), 1607017 (2017). http://doi:10.1002/adma.201607017

[S20] J.S. Moon, J.H. Jang, E.G. Kim, Y.H. Chung, S.J. Yoo, Y.K. Lee, The nature of active sites of Ni_2P electrocatalyst for hydrogen evolution reaction. J. Cat. **326**, 92-99 (2015). http://doi:10.1016/j.jcat.2015.03.012

[S21] L.B. Ma, X.P. Shen, H. Zhou, G.X. Zhu, Z.Y. Ji, K.M. Chen, CoP nanoparticles deposited on reduced graphene oxide sheets as an active electrocatalyst for the hydrogen evolution reaction. J. Mater. Chemi. A **3**(10), 5337-5343 (2015). http://doi:10.1039/c4ta06458e

[S22] Y. Xu, R. Wu, J.F. Zhang, Y.M. Shi, B. Zhang, Anion-exchange synthesis of nanoporous FeP nanosheets as electrocatalysts for hydrogen evolution reaction. Chem. Commun. **49**(59), 6656-6658 (2013). http://doi:10.1039/c3cc43107j
